# The Mediating Role of Eating Attitudes in Sociocultural Attitudes toward the Body in Predicting Obligatory Exercise among Young People: A Polish and Chinese Comparison

**DOI:** 10.3390/nu15040952

**Published:** 2023-02-14

**Authors:** Shuai Guo, Agata Kamionka, Bernadetta Izydorczyk, Malgorzata Lipowska, Sebastian Lizinczyk, Bartosz M. Radtke, Urszula Sajewicz-Radtke, Mariusz Lipowski

**Affiliations:** 1 Faculty of Sport and Leisure, Guangdong Ocean University, Zhanjiang 524000, China; 2 Faculty of Physical Culture, Gdansk University of Physical Education and Sport, 80-336 Gdańsk, Poland; 3 Institute of Psychology, Jagiellonian University, 30-374 Krakow, Poland; 4 Institute of Psychology, University of Gdańsk, 80-309 Gdańsk, Poland; 5 Central Board of Prison Service, Ministry of Justice, 00-950 Warsaw, Poland; 6 Laboratory of Psychological and Educational Tests, 80-239 Gdańsk, Poland; 7 Faculty of Social and Humanities, WSB University in Gdańsk, 80-266 Gdańsk, Poland

**Keywords:** obligatory exercise, eating attitudes, body, mass media, cross-cultural, sociocultural attitudes toward the body

## Abstract

The main aims of this study were to determine which sociocultural predictors of obligatory exercise are universal for young men or women and which are specific to particular cultural conditions (Polish or Chinese culture) and to examine the mediating role of eating attitudes. A cross-sectional study was conducted among Poles (n = 259) and Chinese (n = 208) aged 18 to 30. Descriptive and comparative statistics, Spearman’s rho, and multiple regression analysis were used. The main analyses showed that Internalization—Athlete was a common positive direct predictor of obligatory exercise among young Polish and Chinese women; Information and Internalization—Athlete were only specific direct positive predictors of obligatory exercise in young Chinese men; some variables in eating attitudes mediated the development of obligatory exercise in young Polish and Chinese men and women and indicated that there were cross-cultural differences. In understanding obligatory exercise among young people, attention should be paid to their sociocultural attitudes toward the body and eating, and cultural and gender differences need to be considered.

## 1. Introduction

Regular exercise has been demonstrated to promote physical and mental health and enhance well-being [1,2,3,4]. However, when exercise time and frequency exceed safe upper limits, adverse effects such as skeletal muscle damage, metabolic disturbances, and mood disorders can occur [5,6,7,8]. One of these outcomes is known as obligatory exercise, defined by Polivy [9] as continued participation in physical activity despite the pain, lack of leisure time, interference with work or important relationships, and the social consequences of indulgence. Obligatory exercise has a full range of effects on the exerciser’s body, psychology, work, social relationships, family life, etc. [10,11]. From a global public health perspective, obligatory exercise is more prominent among emerging adults aged 18 to 30 and is becoming a threat to young people’s health in Europe, Asia, and the Americas, including Poland and China [11,12,13,14,15,16,17,18,19]. Sociocultural factors have been shown to play a crucial role in developing obligatory exercise [15]. With the development of modern communication media in the form of the Internet, mass media and public information profoundly influence people’s lives and thoughts. An increasing number of young men and women are engaging in anti-health behaviors, including excessive exercise, dietary restriction, and medication use, to achieve the ideal body image promoted by the media [5,20,21,22]. In the context of globalization, Western culture is widely promoted worldwide, but the cultures of different regions and nationalities remain distinct [23]. Therefore, cross-cultural comparisons of young men and women brought up in different cultures can significantly deepen the diagnostic potential of the sociocultural predictors of obligatory exercise specific to these cultures.

However, cross-cultural and cross-regional studies are rare. This study considers young people from Poland and China who grew up in emerging countries in Europe and Asia. Polish culture is individualistic and Chinese culture is collectivistic [24,25]. However, with the development of globalization, Western culture has been promoted in different countries [26]. Poland and China were extensively influenced by Western culture after the communist period and the start of reform and opening up, respectively [27]. Considering the potential differences and similarities between the cultures of different countries, research on young people in European and Asian countries should consider these potential differences and similarities [28]. For evidence-based practice, measures of obligatory exercise need to be continually validated across different populations from Europe, the Americas, Asia, Africa, and Oceania to expand knowledge about the prevalent and specific sociocultural predictors of obligatory exercise.

In addition, eating attitudes deserve attention in the relationship between sociocultural factors and obligatory exercise in young people. The study by Goodwin et al. [29] confirmed that obligatory exercise is a problematic drive to exercise that is closely associated with eating disorders and will always continue even when exercise is prohibited by injury or illness. A systematic review has shown that up to 85% of people with eating disorders have poor exercise cognition and behaviors [30]. The three-factor model by Thompson et al. explains the strength and nature of the relationship between sociocultural factors (including mass media) and psychological and eating attitudes, and is widely used and validated [31,32,33,34]. The study by Izydorczyk et al. [35] confirmed that the internalization of sociocultural standards of body, Information, Pressures, and Internalization—Athlete were differentially predictive of eating attitudes and behaviors across gender groups. A study on college students’ eating attitudes demonstrated that women felt more pressure from the media about sociocultural standards of the body than men and predicted their restrictive and bulimic behavior [36]. A comparative study on risk factors for eating disorders among Australian and French female university students revealed significant differences in the internalization of body ideals promoted by the mass media and restrictive and bulimic behavior among female university students in the two countries [37]. Furthermore, research based on social comparison theory suggests that obligatory exercise in young people is associated with concerns about appearance and that the internalization of sociocultural standards will predict their obligatory exercise [15,38]. Thus, eating attitudes may mediate the relationship between sociocultural attitudes and obligatory exercise in young people, and there are gender and cultural differences.

**Figure 1:**
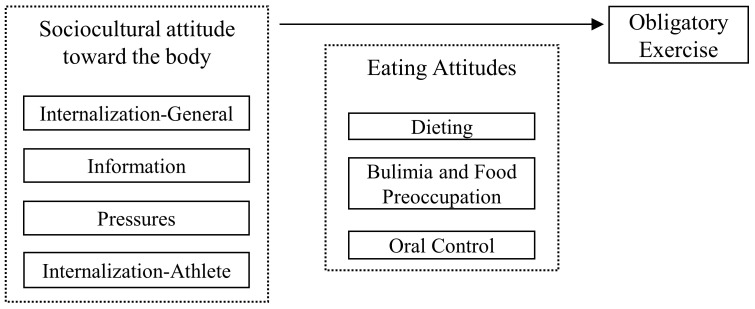
Theoretical model of the research variables (own elaboration).

This study focuses on young men and women in Poland and China. The main aims are to determine which sociocultural predictors of obligatory exercise are universal for young men and women and which are specific to particular cultural conditions (Polish or Chinese culture) and to examine the mediating role of eating attitudes (Figure 1). The study of this issue may extend the scientific field related to physical activity and eating behavior to support young people’s healthy behavioral choices. The authors found existing studies do not compare predictors of eating disorders and obligatory exercise among young people in Poland and China.

## 2. Materials and Methods

### 2.1. Study Design

The data used for this study were part of a large international research project registered in the Protocol Registration and Results System (ClinicalTrials.gov; https://clinicaltrials.gov/ct2/show/NCT04432038). The study was conducted simultaneously in 2021 in four academic cities in Poland and China (Krakow, Gdansk, Beijing, and Zhengzhou). The research team first trained qualified researchers (members of the research team and students of the authors) on research procedures and ethics. The researchers then disseminated information about the possibility of participating in the study among university students in the four cities and sent informed consent forms and online addresses for completing questionnaires via email to those who met the inclusion criteria. At the same time, university students who met the inclusion criteria were asked to help invite their classmates to participate (“snowball sampling technique”). All survey participants provided informed consent and questionnaires.

### 2.2. Participants

Groups were selected by purposive sampling. Participants were recruited using convenience and purposive sampling methods. The following inclusion criteria were used: age (18–30 years), Polish or Chinese nationality and having grown up in the country, no physical disability or physical illness preventing physical activity, not receiving treatment for any eating disorder, and student or graduate in humanities and social sciences. These criteria were validated with the help of a questionnaire, which allowed for the identification of exclusion factors.

The study was planned to cover 300 young Poles and 300 young Chinese. The final study included 303 Poles and 400 Chinese. Due to errors in filling out the questionnaire (incomplete data obtained) and not meeting the inclusion criteria, 38 Poles and 45 Chinese were excluded. The mean age of young Polish men and women was 24.3 (SD = 3.30) and 25.3 (SD = 3.04), respectively; the mean age of young Chinese men and women was 22.0 (SD = 2.61) and 22.1 (SD = 3.11), respectively. The average BMI values for young Polish men and women were 24.3 and 21.5, respectively, and for young Chinese men and women, they were 22.7 and 20.9, respectively; both fell within the normal range of 20 to 25. All respondents were undergraduate and postgraduate students and university graduates currently living in Poland or the surveyed cities in China; 68% were students, and the remainder (32%) were college graduates who were employed at the time of the study; 69% were single and had never married, 21% were married or in a domestic partnership, and 9% were living apart together (LAT). All respondents had no experience as athletes and were not professional physical education learners.

### 2.3. Ethical Approval

The study was conducted following the World Medical Association’s Ethical Guidelines for Research Involving Human Beings (Declaration of Helsinki). The Ethics Committee of the Institute of Psychology, University of Gdansk, Poland (Decision No. 33/2020) approved the protocol of this study of the Research Project. All participants were informed about the research’s purpose and were asked to complete an electronic informed consent form before registering on the project website.

### 2.4. Methods

The independent variable in this study was sociocultural attitudes toward the body. This is defined by Thompson et al. [34] as a four-factor structural variable describing the degree of internalization of sociocultural standards of body and appearance. The dependent variable in this study was obligatory exercise. Based on the study by Ackard et al. [39], this variable was defined as describing attitudes and behaviors associated with exercise. The mediating variable in this study was eating attitudes. This variable was defined as a three-factor structural variable describing eating disorder attitudes and behaviors [40].

The Sociocultural Attitudes Toward Appearance Questionnaire 3 (SATAQ 3) [34] was used to measure the variable of sociocultural attitudes toward the body. The Obligatory Exercise Questionnaire (OEQ) [39] was used to measure the variable of obligatory exercise. The Eating Attitude Test (EAT-26) [40] was used to measure the variable of eating attitudes. In addition, variables such as gender, country of residence, year of birth, weight, and height were also collected. The BMI was obtained by dividing the body weight in kilograms by the square of the height in meters.

#### 2.4.1. The Sociocultural Attitudes toward Appearance Questionnaire 3 (SATAQ 3)

This study used the Sociocultural Attitudes Toward Appearance Questionnaire 3 (SATAQ 3) by Thompson et al. [34] with a Polish adaptation by Izydorczyk and Lizińczyk [41] and a Chinese adaptation by Jackson and Chen [42]. The SATAQ 3 consists of 30 questions, including four subscales: Internalization—General (assesses the extent to which general sociocultural standards regarding the body and appearance are internalized; includes nine items), Information (measures the frequency of seeking information about sociocultural standards of the body and appearance; includes nine items), Pressures (assesses the perceived pressure of sociocultural standards regarding the body; includes seven items), and Internalization—Athlete (measures the extent to which the ideal of the athletic body is internalized; includes five items). Participants completed the SATAQ 3 questionnaire by marking their answers on a 5-point Likert scale. The total score for each subscale was calculated, with higher scores indicating higher internalization or acceptance. The Cronbach’s alpha coefficients for the four subscales are as follows: Internalization—General (0.937 in Polish groups, 0.941 in Chinese groups), Information (0.899 in Polish groups, 0.922 in Chinese groups), Pressures (0.956 in Polish groups, 0.897 in Chinese groups), Internalization—Athlete (0.871 in Polish groups, 0.841 in Chinese groups).

#### 2.4.2. The Obligatory Exercise Questionnaire (OEQ)

Thompson and Pasman’s [39] Obligatory Exercise Questionnaire (OEQ) was used. The Polish and Chinese versions of the questionnaire were translated using standard forward–backward translation procedures. The OEQ contains 20 items that measure attitudes and activities related to exercise. Respondents completed the questionnaire by marking their answers on a 4-point Likert scale. Higher total OEQ scores indicated that they were more likely to undertake obligatory exercise. The Cronbach’s alpha coefficients for the OEQ were as follows: Polish groups = 0.873, Chinese groups = 0.873.

#### 2.4.3. The Eating Attitude Test (EAT-26)

This study used Thompson et al.’s Eating Attitude Test (EAT-26) with a Polish adaptation by Wlodarczyk-Bisaga and Dolan [43] and a Chinese adaptation by Lee and Lee [44]. The EAT-26 consists of 26 items and contains three subscales: Dieting (assesses attitudes and behaviors that are focused on being thin and avoiding fattening foods; 13 items), Bulimia and Food Preoccupation (assesses overeating, loss of control overeating, and subordination of thoughts and behaviors to food; 6 items), Oral Control (assesses self-control over diet and perceived pressure to gain weight; 7 items). Participants completed the EAT-26 questionnaire by marking their answers on a 6-point Likert scale. Higher scores on the total EAT-26 and the three subscales indicated a greater likelihood of eating attitudes and behavioral disorders. The Cronbach’s alpha coefficients for the three subscales were as follows: Dieting (0.821 for the Polish group, 0.866 for the Chinese group), Bulimia and Food Preoccupation (0.906 for the Polish group, 0.929 for the Chinese group), and Oral Control (0.895 for the Polish group, 0.878 for the Chinese group).

### 2.5. Statistical Methods

The data were analyzed in Excel (Microsoft Office 365) and IBM SPSS (Statistical Package for the Social Sciences) Statistics 26 according to the research objectives and research questions. The statistical analysis stages were as follows: 

Stage 1—Descriptive statistics. Cross-cultural similarities and differences between Polish and Chinese young men and women in terms of sociocultural attitudes toward the body, eating attitudes, and obligatory exercise were elucidated by measuring the mean, quartile, etc., of each variable in the study model. 

Stage 2—Assessing between-group differences of all variables. For the purposes of further statistical analysis, the variables were tested for normality of distribution using the Shapiro–Wilk test. The obtained results indicate that the variables do not meet the conditions of the normal distribution; therefore, non-parametric tests (Mann–Whitney U test) were used for further analysis. The Mann–Whitney U test was used to measure the significance of the differences between the Polish and Chinese groups.

Stage 3—Measuring the strength of relationships between variables in the Polish and Chinese groups. The significance of the difference in the strength of the relationship and the strength of the correlation between the variables in the Polish and Chinese groups was measured using Spearman’s rank correlation coefficient.

Stage 4—Measuring the strength of the relationship between independent and dependent variables using multiple regression analysis and testing the mediating role of eating attitudes. This phase aimed to find predictors of obligatory exercise among young men and women in Poland and China. Calculations were performed using the PROCESS macro for SPSS [45].

The study presents an integrated model of the hypothesized relationship between variables to explain the direct predictive role of sociocultural factors and the mediating role of eating attitudes factors in explaining the emergence of obligatory exercise. The direct and indirect effects were tested using Model 4 in the PROCESS macro of SPSS. The significance of the indirect effects was tested using bootstrapping, and a bootstrap sample of 5000 was used to model the data distribution better. The confidence interval (CI) was 95%. The effect is not considered significant if the confidence interval contains a zero value. Only unstandardized estimates can be calculated. The hypothesized relationships for validation include only those variables for which there is a significant relationship. 

## 3. Results

### 3.1. Characteristics of Sociocultural Attitudes toward the Body, Eating Attitudes, and Obligatory Exercise in Young Polish and Chinese (Differences between the Groups)

Table 1 shows the results of the comparative analysis of the quartiles of all variables for the Polish and Chinese male and female groups. It can be seen that there are significant differences between young Polish men and women and young Chinese men and women in some variables.

In terms of sociocultural attitudes toward the body, young Polish and Chinese women show significant differences in all variables except the Pressures variable, and young Polish and Chinese men show significant differences in all variables except the Internalization—Athlete variable. Young Polish women are significantly lower than young Chinese women in the Internalization—General and Information variables and significantly higher than young Chinese women in the Internalization—Athlete variable. On the other hand, young Polish men show significantly lower levels of Internalization—General, Information, and Pressures variables than young Chinese men. 

In terms of eating attitudes, there were significant differences between young Polish and Chinese women in the Dieting, Bulimia and Food Preoccupation, and Oral Control variables, with young Chinese women showing higher levels than young Polish women, except for Dieting. There were significant differences between young Polish and Chinese men in the Bulimia and Food Preoccupation and Oral Control variables, with young Polish men showing lower levels than young Chinese men. 

The comparative analysis of the quartiles also shows significant differences between young Polish men and women in obligatory exercise. The mean level of obligatory exercise for young Polish women was significantly higher than that of young Chinese women. The mean level of obligatory exercise for young Polish men was significantly lower than that of young Chinese men. 

### 3.2. Relationship between Studied Variables among Young Polish and Chinese Men and Women

Table 2 shows the statistical analysis results of all variables for the different groups. It can be seen that the partial variables of sociocultural attitudes toward the body were significantly associated with the partial variables of eating attitudes and obligatory exercise among young Polish and Chinese men and women; the partial variables of eating attitudes were significantly associated with obligatory exercise. The correlations between the study model variables were both similar and different between young Polish and Chinese men and women. In addition, the significance of the correlations between the variables in the study model offers the possibility of analyzing the direct and indirect predictive effects of sociocultural attitudes toward the body.

### 3.3. Predictors of Obligatory Exercise among Young Men and Women in Poland and China

**Figure 2:**
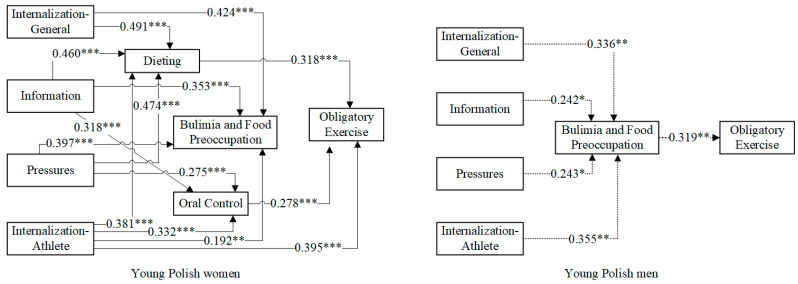
Sociocultural (Internalization—General, Information, Pressures, and Internalization—Athlete) and eating attitude (Dieting, Bulimia and Food Preoccupation, and Oral Control) predictors of obligatory exercise among young Polish men and women (n = 265). Note: Non-standardized estimates are presented—* *p* < 0.05, ** *p* < 0.01, *** *p* < 0.001—to improve the readability of the presentation; only paths with significance levels <0.05 were retained.

**Figure 3:**
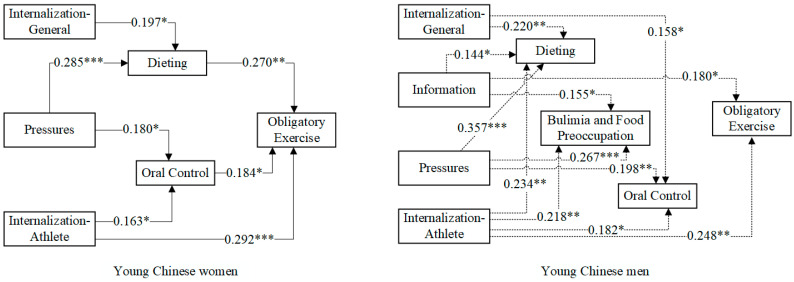
Sociocultural (Internalization—General, Information, Pressures, and Internalization—Athlete) and eating attitudes (Dieting, Bulimia and Food Preoccupation, and Oral Control) predictors of obligatory exercise among young Chinese men and women (n = 355). Note: Non-standardized estimates are presented—* *p* < 0.05, ** *p* < 0.01, *** *p* < 0.001—to improve the readability of the presentation; only paths with significance levels <0.05 were retained.

Figure 2 and Figure 3 show the predictive effects of the four variables on social attitudes toward the body for young Polish and Chinese men and women, respectively, with only paths with significance levels <0.05 retained to improve the readability of the presentation. Table 3 shows the model estimates for the three variables of eating attitudes as mediators of the four variables on sociocultural attitudes toward the body as predictors of obligatory exercise. The group effect is insignificant if a 0 value is included between the Lower 95% CI and the Upper 95% CI.

From Figure 2, we can see the following:

Among young Polish women, all four variables on sociocultural attitudes toward the body were significant in predicting the path of the three variables on eating attitudes, except for the path of the Internalization—General variable predicting the Oral Control variable. The Internalization—Athlete variable was a predictor of obligatory exercise. In addition, the Dieting and Oral Control variables were predictors of obligatory exercise.

Among young Polish men, all four variables on sociocultural attitudes toward the body were predictors of the Bulimia and Food Preoccupation variable. Furthermore, the Bulimia and Food Preoccupation variable was a predictor of obligatory exercise.

From Figure 3, the following can be seen:

Among young Chinese women, the Internalization—General variable was a predictor of the Dieting variable, the Pressures variable was a predictor of the Dieting and Oral Control variables, and the Internalization—Athlete variable was a predictor of the Oral Control variable. The Internalization—Athlete variable was a predictor of obligatory exercise. Finally, Dieting variables and Oral Control variables were predictors of obligatory exercise.

In young Chinese men, the Internalization—General variable was a predictor of the Dieting and Oral Control variables, the Information variable was a predictor of the Dieting and Bulimia and Food Preoccupation variables, and the Pressures and Internalization—Athlete variables were predictors of all three variables of eating attitudes. The Pressures variable and the Internalization—Athlete variable were both predictors of the three eating attitudes variables. The Information variable and the Internalization—Athlete variable were predictors of obligatory exercise.

In combination with Table 3, the following can be observed:

The level of internalization of athletic body ideals promoted by mass media directly predicted obligatory exercise among young Polish women, young Chinese women, and young Chinese men. The frequency of seeking mass media information on sociocultural standards of body and appearance directly predicted obligatory exercise only among young Chinese men. Among Polish and Chinese young men and women, acceptance of the general sociocultural standards of body and appearance promoted by the mass media and the level of perceived pressure from the sociocultural standards of body and appearance promoted by the mass media were not direct predictors of obligatory exercise.

Among young Polish women, all three remaining variables of sociocultural attitudes toward the body, except for the Internalization—Athlete variable, indirectly predicted obligatory exercise through the Dieting variable. All three remaining variables on sociocultural attitudes of the body, except for the Internalization—General variable, indirectly predicted obligatory exercise through the Oral Control variable.

Among young Chinese women, only the Internalization—General variable and the Pressures variable indirectly predicted obligatory exercise through the Dieting variable.

Among young Polish men, all four variables of sociocultural attitudes toward the body indirectly predicted obligatory exercise through the Bulimia and Food Preoccupation variable.

Among young Chinese men, no eating attitudes variables could mediate sociocultural attitudes toward the body to predict obligatory exercise.

## 4. Discussion

### 4.1. Characteristics of Sociocultural Attitudes toward the Body, Eating Attitudes, and Obligatory Exercise in Young Polish and Chinese

The results of the study indicate that there is no significant difference between young Polish and Chinese women in terms of perceived pressure to sociocultural standards regarding the body promoted by mass media, and no significant difference between young Polish and Chinese men in terms of recognition and acceptance of the athletic body ideal promoted by mass media. Other sociocultural attitudes toward the body differed significantly between young Polish and Chinese people in a same-gender comparison. Compared to young Chinese women, young Polish women showed a higher acceptance of the athletic body ideal promoted by mass media, while they were less receptive to the general sociocultural standards regarding the body promoted by mass media and less frequently sought information about sociocultural standards regarding the body and appearance promoted by mass media. Compared to young Chinese men, young Polish men were less receptive to general sociocultural standards regarding the body promoted by the mass media, less frequently sought information about sociocultural standards regarding the body and appearance promoted by the mass media, and felt less pressure about sociocultural standards regarding the body and appearance promoted by the mass media. The findings of several cross-cultural studies of sociocultural attitudes toward the body confirm these results. A comparative study of Polish and Vietnamese university students proved that, due to cultural differences, Vietnamese female university students focus on quietness and submissiveness and favor bodies that conform to general sociocultural norms; Vietnamese male university students focus on muscle, strength, and male-dominated social roles; and Polish young men and women focus on personality, personal experience, and personal physical attractiveness [46]. A comparative study of young Polish and Japanese women also proves that Japanese women are more eager to seek information about sociocultural standards of the body and appearance promoted by the mass media [47]. Furthermore, research on the sociocultural attitudes toward the body of young Chinese people proves that young Chinese men show higher levels of recognition of general sociocultural standards regarding the body, frequency of seeking information on sociocultural standards regarding the body promoted by the mass media, and perceived pressure from sociocultural attitudes toward the body and appearance in the mass media [42,48]. Although there is no direct comparative study of young people in Poland and China, Vietnam, Japan, and China are part of the same East Asian cultural sphere and are deeply related in terms of history and cultural environment [49]. These findings are further supported by the fact that more factors of sociocultural attitudes toward the body were shown to be higher among the young Chinese men and women in this study than among the young Polish men and women.

In terms of eating attitudes, the results of this study indicate that both Polish and Chinese men are very concerned about thinness and the avoidance of fatty foods, but in other areas, young Polish and Chinese people show significant differences. Compared to young Chinese women, young Polish women were more concerned with thinness and the avoidance of fatty foods, showed lower levels of bulimia and uncontrolled eating, and had slightly lower levels of self-control overeating requirements and perceived pressure to gain weight. Compared to young Chinese men, young Polish men showed lower levels of bulimia and uncontrolled eating, as well as lower self-control overeating requirements and perceived pressure to gain weight. A study by Li et al. [50] on eating attitudes in Chinese and American female university students demonstrated that European and American women in individualistic societies actively pursue thinness, compared to Chinese female university students who do not perceive themselves as being larger and needing to lose weight and diet. Izydorczyk et al. [28] showed that young Polish women were significantly higher than their Vietnamese peers in monitoring their weight and dieting overall, while they were significantly lower than their Vietnamese peers in binge-eating behavior. These studies, to some extent, support the findings of this study on Dieting and Bulimia and Food Preoccupation among young Polish and Chinese women. Moreover, sociologically and anthropologically, eating has a vital social function in Chinese culture, and Chinese adults may overeat or feel pressure from others to gain weight in order to maintain relationships and celebrate important events [51,52]. However, the eating patterns of European and American adults with individualistic cultural values are more akin to a “functional diet”, with a focus on personal experience and achieving maximum results (e.g., providing nutrition, satisfying hunger) at minimal cost (e.g., saving time) [53,54]. This partly supports the fact that young Chinese men and women in this study performed higher than young Polish men and women in terms of uncontrolled eating and feeling pressure from others to gain weight.

Furthermore, the findings of this study suggest that compared to young Chinese people of the same gender, young Polish women are more likely to undertake obligatory exercise, while young Polish men are less likely to do so. Although no comparative studies on obligatory exercise between Polish and Chinese young people were found, a study on the physical activity of French and Japanese adults confirmed that adult physical activity varies by gender and culture and that the global wave of Western sports and physical culture has not turned Japanese sports into an exact replica of Western sports [55]. Some studies also demonstrated that a solid sociocultural background influences obligatory exercise through body ideals [14,16,56]. The differences in obligatory exercise exhibited by young Polish and Chinese men and women in this study may be due to differences in their sociocultural attitudes toward the body. This provides support for the analysis of the subsequent questions in this study.

### 4.2. Sociocultural Attitudes toward the Body Predicting Obligatory Exercise with a Comparison between Young Polish and Chinese

#### 4.2.1. Sociocultural Attitudes toward the Body as a Direct Predictor of Obligatory Exercise

This study aimed to determine which sociocultural predictors of obligatory exercise are universal for young men or women and which are specific to particular cultural conditions (Polish or Chinese culture) and to examine the mediating role of eating attitudes. The results of this study show that Internalization—Athlete in sociocultural attitudes toward the body is a common direct positive predictor of obligatory exercise among young women in Poland and China. Importantly, this study suggests that the acceptance of the athletic body ideal promoted by the mass media among young women will directly and positively predict their obligatory exercise. A study by Homan [57] on female college students indicated that female college students’ internalization of athletic ideals predicted their obligatory exercise. Bell et al. [58] and Girard et al. [59] confirmed that the internalization of athletic ideals directly predicted obligatory exercise in adult women. The results of the present study further support these studies.

On the other hand, this study’s results confirm the existence of specific sociocultural predictors of obligatory exercise in young Chinese men. Information and Internalization—Athlete in sociocultural attitudes toward the body were direct positive predictors of obligatory exercise in young Chinese men. In contrast, this was not the case among young Polish men. Research on the body image of young Polish men also confirmed that the frequency with which young Polish men seek information about the body and appearance in mass media, perceived pressure to look good, and endorsement of athletic body ideals do not directly predict their physical behavior [35,60,61]. However, there is a paucity of existing research on the factors influencing obligatory exercise in young Chinese men, making the results of this study significant. Young Chinese males grow up in a climate of collectivist cultural values, where their attitudes and behaviors are easily influenced by their social environment and are more concerned with male-dominated social status as well as muscle and strength [46,62]. Combined with social comparison theory [63], to maintain their male-dominated social role, the heightened focus on information about sociocultural standards of the body promoted by the mass media and higher acceptance of the athletic body ideal promoted by the mass media among young Chinese men may translate into more frequent and intense exercise.

#### 4.2.2. Eating Attitudes as Mediators between Sociocultural Attitudes toward the Body and Obligatory Exercise

The results of this study also suggest that eating attitudes mediate the relationship between sociocultural attitudes toward the body and obligatory exercise and that there are general mediators that apply to both Poland and China and mediators that apply only to a specific culture (Polish or Chinese culture), and that there are clear gender differences. Among Polish and Chinese young women, Internalization—General and Pressures in sociocultural attitudes toward the body indirectly and positively predict obligatory exercise through Dieting in eating attitudes. This demonstrates that the higher the level of acceptance and perceived pressure of the general sociocultural standards regarding the body promoted by the mass media among young women, the more concerned they are about being thin and avoiding fatty foods, and the more likely they are to engage in obligatory exercise. Some studies also confirm that, with the global spread of Western aesthetic standards of beauty based on thinness, the sociocultural acceptance of this and the perceived pressure on young women will translate into body dissatisfaction, which will lead to an increase in their dieting rates and excessive exercise [15,57,64]. Although few studies have examined the same mediating role of eating disorders as this study, the above findings still support the results of this study to some extent. 

In addition, the results of this study show that the more frequently young Polish women seek information about sociocultural standards of body and appearance promoted by the mass media, the more they focus on thinness and avoiding fatty foods, and the more likely they are to engage in obligatory exercise. The frequency with which young Polish women seek information about sociocultural standards of body and appearance promoted by the mass media, the perceived pressure of sociocultural body standards promoted by the mass media, and the recognition of the ideal of the athletic body promoted by the mass media indirectly positively influence their obligatory exercise through dietary self-control and pressure to gain weight. However, this was not the case for young Chinese women. Young Polish men’s acceptance of prevailing sociocultural standards regarding the body promoted by the mass media, the frequency of seeking information about sociocultural standards regarding the body and appearance promoted by the mass media, the pressure to meet sociocultural standards regarding the body promoted by the mass media, and the endorsement of the ideal of the athletic body promoted by the mass media indirectly and positively influence their obligatory exercise through overeating and uncontrolled eating. However, this was not the case among young Chinese men. This may be due to cultural differences between Poland and China. Polish culture is individualistic; Polish people focus on individual characteristics and personal experiences and insist on achieving their personal goals and aspirations through practical behavior [46,65]. Studies by Brytek-Matera et al. [66], Gramaglia et al. [67], and Izydorczyk et al. [68] demonstrated the frequency with which young Polish women seek out mass media information on sociocultural standards of body and appearance and the perceived stress as predictors of their drastic dieting and focus on thinness. According to the culture of individualism, the pursuit of thinness and dieting among young Polish women may lead them to exercise more frequently or at higher intensities. A study of predictors of bulimia behavior in young Polish demonstrated that the higher the level of recognition and acceptance of general sociocultural attitudes toward body and appearance promoted by the mass media among young men, the higher the incidence of bulimia or uncontrolled eating among them [35]. According to social comparison theory [63], under the influence of sociocultural attitudes toward body and appearance, young Polish men may choose to exercise more frequently and with greater intensity in order to counteract the adverse effects of bulimic eating on one’s body and mind, which may lead to an increased incidence of obligatory exercise among them. On the other hand, a literature review by Pike and Dunne [69] on the rise of eating disorders in Asia suggests that the development of eating disorders and disordered eating attitudes and behaviors is closely related to urbanization and industrialization, with some cases of eating disorders reported in Japan, yet disordered eating attitudes and behaviors are rarely reported in other countries and regions, including mainland China. Thus, symptoms such as binge eating and uncontrolled eating are less reported among young people in mainland China, which may be why no eating attitude mediators similar to those found in young Polish men and women were found in this study. As the economy develops, it is questionable whether eating attitude mediators that predict current mandatory exercise among young Polish men and women will emerge in China. This study team will also continue to follow up.

The findings of the resulting analysis suggest significant cultural differences in the predictors of obligatory exercise for young men and women. Both common and culture-specific predictors exist. The present study further confirms existing cross-cultural related research and contributes to exploring global universal or culture-specific predictors of obligatory exercise in young people. Preventive interventions for obligatory exercise in young people should pay attention to the sociocultural standards regarding the body promoted by the mass media, with particular attention to the mediating role of eating attitudes in the development of obligatory exercise and the various effects of different sociocultural contexts and different genders. The authors recommend including such interventions in health universities and national fitness programs. The practical implications of this study revolve around professionals involved in physical activity and wellness programs for university students. In the practices of educators, physicians, psychologists, and other specialists who support the healthy development of young people, measures of sociocultural attitudes toward the body and eating attitudes may be necessary.

### 4.3. Limitations of the Study

This study presents a rare, even unique, opportunity to make comparisons between two different cultures. However, it also has notable limitations. Firstly, the study’s sample size, although sufficient for statistical analysis, was not large. Secondly, although the paper focuses on obligatory exercise perceptions rather than exercise behavior, the lack of measures of exercise behavior may be a limitation. The research analysis provided an opportunity to compare two distinctly different cultures and yielded new results lacking in the literature. Future research will consider obligatory exercise behavior in conjunction with forced exercise perceptions and work toward larger sample sizes. Meanwhile, a long-term longitudinal study will be considered that will continue to follow the comparison of predictors of compulsive exercise among Polish and Chinese youth.

## 5. Conclusions

The comparison between young Polish and Chinese people showed that the variables studied differed in terms of culture and gender. Young Polish women and young Chinese men showed a higher intensity in obligatory exercise (dependent variable). Young Chinese men and women showed a higher intensity in Internalization—General and Information (independent variables), young Chinese men showed a higher intensity in Pressures (independent variable), and young Polish women showed a higher intensity in Internalization—Athlete (independent variable). In terms of eating attitudes (mediating variable), young Polish men showed a higher intensity in Dieting, while young Chinese women and men showed a higher intensity in Bulimia and Food Preoccupation and Oral Control. 

The findings suggest that Internalization—Athlete in sociocultural attitudes toward the body is a common positive direct predictor of obligatory exercise among young Polish and Chinese women. At the same time, the study found that Information and Internalization—Athlete in sociocultural attitudes toward the body were only specific direct positive predictors of obligatory exercise in young Chinese men, but not in young Polish men.

It is worth noting that some of the variables in eating attitudes mediate the relationship between the independent variable and the dependent variable, and that there are common mediating variables that apply to both Polish and Chinese women, and specific mediating variables that apply only to young Polish men or women.

## Figures and Tables

**Table 1:** Comparative analysis of the young Polish and Chinese male and female groups according to the variables included in the research model.

Variables	Female							Male						
	Polish (N = 186)			Chinese (N = 161)				Polish (N = 79)			Chinese (N = 194)			
	Q1	Median	Q3	Q1	Median	Q3	*p*	Q1	Median	Q3	Q1	Median	Q3	*p*
Internalization—General	18	26	31	24	27	31	0.003	15	19	24	25	27	30	<0.001
Information	17	26	30	26	27	31	<0.001	11	16	21	26	27	30	<0.001
Pressures	13	21	27	17	20	23	0.507	7	11	15	17	20	21	<0.001
Internalization—Athlete	13	19	22	13	15	18	<0.001	13	16	19	14	15	18	0.448
Dieting	33	41	47	27	35	41	<0.001	25	31	38	25	32	40	0.81
Bulimia and Food Preoccupation	9	11	13	12	14	17	<0.001	7	9	12	10	13	16	<0.001
Oral Control	14	17	20	16	18	20	0.047	11	14	18	15	18	21	<0.001
Obligatory Exercise	43	52	57	40	46	53	<0.001	40	47	54	44	51	58	0.005

The significance threshold was set at 0.05; N, number of people; Q1, 25% Quartile; Q2, 75% Quartile.

**Table 2:** Correlation analysis for all variables for the groups of Polish and Chinese.

Variables		Internalization—General		Information		Pressures		Internalization—Athlete		Obligatory Exercise	
		Polish	Chinese	Polish	Chinese	Polish	Chinese	Polish	Chinese	Polish	Chinese
Dieting	female	0.447 **	0.173 *	0.428 **	0.068	0.448 **	0.304 **	0.365 **	0.062	0.450 **	0.258 **
	male	0.129	0.245 **	0.182	0.206 **	0.138	0.382 **	0.151	0.226 **	0.380 **	0.143 *
Bulimia and Food Preoccupation	female	0.372 **	0.010	0.359 **	−0.113	0.414 **	0.106	0.272 **	0.005	0.298 **	0.185 *
	male	0.326 **	0.189 **	0.230 *	0.182 *	0.255 *	0.272 **	0.358 **	0.197 **	0.258 *	0.056
Oral Control	female	0.077	0.150	0.342 **	0.099	0.276 **	0.217 **	0.392 **	0.167 *	0.412 **	0.245 **
	male	0.172	0.190 **	0.107	0.136	0.175	0.252 **	0.191	0.179 *	−0.029	0.095
Obligatory Exercise	female	0.267 **	0.020	0.392 **	0.057	0.318 **	0.076	0.540 **	0.314 **	1.000	1.000
	male	0.231 *	0.165 *	0.226 *	0.254 **	0.041	0.188 **	0.318 **	0.264 **	1.000	1.000

* *p* < 0.05; ** *p* < 0.01

**Table 3:** Model estimates for direct prediction of four variables on sociocultural attitudes toward the body and indirect prediction of obligatory exercise through three variables on eating attitudes.

		Estimates	SE	*p*	Lower 95% CI	Upper 95% CI
	**Direct effects**					
Young Polish women	Internalization—Athlete	0.395	0.067	<0.001	0.264	0.527
Young Chinese women	Internalization—Athlete	0.292	0.075	<0.001	0.143	0.440
Young Chinese men	Information	0.180	0.072	0.013	0.038	0.322
	Internalization—Athlete	0.248	0.072	0.001	0.106	0.391
	**Indirect effects via Dieting**					
Young Polish women	Internalization—General	0.156	0.054		0.056	0.268
	Information	0.118	0.047		0.030	0.213
	Pressures	0.133	0.049		0.042	0.237
	Internalization—Athlete	0.062	0.038		−0.011	0.142
Young Chinese women	Internalization—General	0.053	0.027		0.005	0.111
	Pressures	0.059	0.029		0.010	0.121
	**Indirect effects via Bulimia and Food Preoccupation**					
Young Polish men	Internalization—General	0.107	0.048		0.023	0.211
	Information	0.078	0.036		0.015	0.157
	Pressures	0.092	0.043		0.016	0.183
	Internalization—Athlete	0.099	0.045		0.019	0.198
	**Indirect effects via Oral Control**					
Young Polish women	Information	0.088	0.034		0.033	0.166
	Pressures	0.081	0.033		0.028	0.153
	Internalization—Athlete	0.066	0.031		0.016	0.137
Young Chinese women	Pressures	0.028	0.020		−0.002	0.073
	Internalization—Athlete	0.030	0.021		−0.003	0.076

Non-standardized estimates are presented; non-significant effects are depicted in bold; the indirect effect is not considered significant if the confidence interval contains a zero value.

## Data Availability

The data of this study are available from the corresponding author upon request.
